# Dr. Torrie on Poisoning by Cantharides

**Published:** 1826-07

**Authors:** 


### POISONING BY CANTHARIDES.

On the second of May, 1825, Dr. Torrie was called to a man who complained of much heat and pain in the region of the stomach, which was in a very irritable state. The tongue, throat, and gums were ulcerated—copious discharge of saliva—frequent inclination to make water—great pain in doing so—thirst—pulse small, and 100 in the minute. On inquiry it was found that a man with whom he had been drinking, two days previously, had given him a drachm of the powdered lytta in some ale! Fortunately the poor fellow vomited a good deal soon after taking the poison, and doubtless by that means threw off a part of the powder. He soon recovered by diluents, opium, camphor, and castor oil.

At the trial of the man who administered the lytta, there was, as usual, a clashing of medical evidence. One medical man stated that he had been in the habit of giving *ten grains* of the powdered lytta "as a single dose." This was Dr. Dyce of Aberdeen. We would be glad to ask Dr. Dyce, on what occasions and for what complaints he administered such doses of the lytta? We would also venture to dissuade medical men against the practice of embarking on opposite sides of criminal accusations, to be made the tools of lawyers—to embarrass Juries—to give vent to their own crude and jarring *opinions*—and to bring disgrace on medical science.

